# Taking the metabolic pulse of the world’s coral reefs

**DOI:** 10.1371/journal.pone.0190872

**Published:** 2018-01-09

**Authors:** Tyler Cyronak, Andreas J. Andersson, Chris Langdon, Rebecca Albright, Nicholas R. Bates, Ken Caldeira, Renee Carlton, Jorge E. Corredor, Rob B. Dunbar, Ian Enochs, Jonathan Erez, Bradley D. Eyre, Jean-Pierre Gattuso, Dwight Gledhill, Hajime Kayanne, David I. Kline, David A. Koweek, Coulson Lantz, Boaz Lazar, Derek Manzello, Ashly McMahon, Melissa Meléndez, Heather N. Page, Isaac R. Santos, Kai G. Schulz, Emily Shaw, Jacob Silverman, Atsushi Suzuki, Lida Teneva, Atsushi Watanabe, Shoji Yamamoto

**Affiliations:** 1 Scripps Institution of Oceanography, University of California San Diego, La Jolla, California, United States of America; 2 The Rosential School of Marine & Atmospheric Science, University of Miami, Miami, Florida, United States of America; 3 Department of Global Ecology, Carnegie Institution for Science, Stanford, California, United States of America; 4 Bermuda Institute of Ocean Sciences, St. George’s, Bermuda; 5 University of Southampton, Southampton, United Kingdom; 6 Atlantic Oceanographic and Meteorological Laboratory, NOAA, Miami, Florida, United States of America; 7 Cooperative Institute for Marine and Atmospheric Studies, Rosenstiel School of Marine and Atmospheric Science, University of Miami, Miami, Florida, United States of America; 8 Department of Marine Sciences, University of Puerto Rico, Mayagüez, Puerto Rico; 9 Department of Earth System Science, Stanford University, Stanford, California, United States of America; 10 Institute of Earth Sciences, The Hebrew University, Jerusalem, Israel; 11 Centre for Coastal Biogeochemistry Research, Southern Cross University, Lismore, New South Wales, Australia; 12 CNRS-INSU, Laboratoire d’Océanographie de Villefranche, Villefranche-sur-mer, France; 13 Sorbonne Universités, UPMC Univ Paris 06, Observatoire Océanologique, Villefranche-sur-mer, France; 14 Institute for Sustainable Development and International Relations, Sciences Po, Paris, France; 15 National Oceanic and Atmospheric Administration Ocean Acidification Program, Silver Spring, Maryland, United States of America; 16 Department of Earth and Planetary Science, The University of Tokyo, Tokyo, Japan; 17 Smithsonian Tropical Research Institute, Balboa, Ancon, Republic of Panama; 18 National Marine Science Centre, Southern Cross University, Coffs Harbour, New South Wales, Australia; 19 School of Marine Science and Ocean Engineering, University of New Hampshire, Durham, New Hampshire; 20 Department of Biology, California State University, Northridge, California, United States of America; 21 National Institute of Oceanography, Haifa, Israel; 22 Geological Survey of Japan, National Institute of Advanced Industrial Science and Technology, Tsukuba, Ibaraki, Japan; 23 Conservation International, Center for Oceans, Honolulu, Hawaii, United States of America; 24 Department of Mechanical and Environmental Informatics, Tokyo Institute of Technology, Meguro, Tokyo, Japan; King Abdullah University of Science and Technology, SAUDI ARABIA

## Abstract

Worldwide, coral reef ecosystems are experiencing increasing pressure from a variety of anthropogenic perturbations including ocean warming and acidification, increased sedimentation, eutrophication, and overfishing, which could shift reefs to a condition of net calcium carbonate (CaCO_3_) dissolution and erosion. Herein, we determine the net calcification potential and the relative balance of net organic carbon metabolism (net community production; NCP) and net inorganic carbon metabolism (net community calcification; NCC) within 23 coral reef locations across the globe. In light of these results, we consider the suitability of using these two metrics developed from total alkalinity (TA) and dissolved inorganic carbon (DIC) measurements collected on different spatiotemporal scales to monitor coral reef biogeochemistry under anthropogenic change. All reefs in this study were net calcifying for the majority of observations as inferred from alkalinity depletion relative to offshore, although occasional observations of net dissolution occurred at most locations. However, reefs with lower net calcification potential (i.e., lower TA depletion) could shift towards net dissolution sooner than reefs with a higher potential. The percent influence of organic carbon fluxes on total changes in dissolved inorganic carbon (DIC) (i.e., NCP compared to the sum of NCP and NCC) ranged from 32% to 88% and reflected inherent biogeochemical differences between reefs. Reefs with the largest relative percentage of NCP experienced the largest variability in seawater pH for a given change in DIC, which is directly related to the reefs ability to elevate or suppress local pH relative to the open ocean. This work highlights the value of measuring coral reef carbonate chemistry when evaluating their susceptibility to ongoing global environmental change and offers a baseline from which to guide future conservation efforts aimed at preserving these valuable ecosystems.

## Introduction

The health of coral reefs is declining globally due to human induced environmental changes [1–4]. However, individual coral reefs may exhibit different susceptibilities and resistance to environmental change that are dependent on a number of factors such as community composition, reef biogeochemistry, environmental and oceanographic properties, and pressure from local human activities [5–8]. Arguably, one of the most important determinants of overall reef function is the construction and maintenance of calcium carbonate (CaCO_3_) reef structure, which is vital to the myriad of ecosystem services that coral reefs provide [3, 9, 10]. Ocean acidification is expected to eventually shift reefs from a state of net CaCO_3_ precipitation to net dissolution through a reduction in seawater pH and aragonite saturation state (Ω_a_) [11, 12]. Other threats such as coral bleaching, overfishing, and eutrophication could exacerbate the loss of CaCO_3_ structure and habitats via the loss of coral cover and shifts towards algal dominated systems [3, 6]. Consequently, resolving whether coral reef ecosystems will maintain net calcification under future environmental change is perhaps one of the most urgent coral reef research questions that needs to be addressed [13].

While diel and seasonal variations in open ocean surface seawater carbonate chemistry are relatively modest compared to future changes anticipated from ocean acidification, the biogeochemical processes and high metabolic rates of coral reef communities (i.e., the balance of photosynthesis, respiration, calcification, and CaCO_3_ dissolution) can drive dramatic changes in the carbonate chemistry of seawater overlying reef ecosystems on these timescales (e.g., [14–17]). Field observations and numerical modeling results have demonstrated that ocean acidification will interact with local reef biogeochemistry, leading to enhanced variability in future seawater pH due to a reduction in seawater buffering capacity [18, 19]. It has recently been proposed that these biogeochemical processes could locally counteract or exacerbate ocean acidification through changes in seawater pH and Ω_a_ [20–24]. While it is clear that pH will change differently in reefs across the globe due to ocean acidification [18], it is not fully understood whether marine organisms and communities will mainly respond to a reduction in average pH, the occurrence of extreme pH values, changes in pH variability, or some combination [25–28]. Therefore, to gain a better understanding of how coral reefs will respond to ocean acidification it is important to understand how seawater carbonate chemistry currently varies within individual coral reefs, largely because this will affect how local chemistry changes in response to global perturbations.

**Fig 1:**
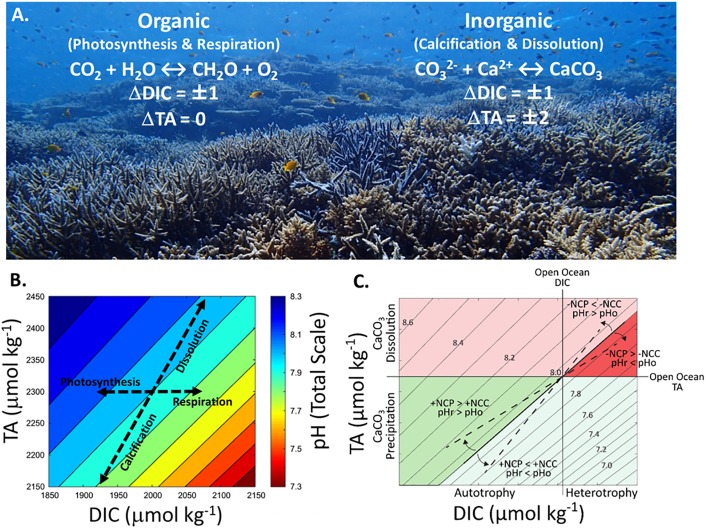
The dominant metabolic processes on coral reefs and their influence on seawater total alkalinity (TA), dissolved inorganic carbon (DIC), and pH. (A) The organic carbon cycle (NCP) is dominated by photosynthesis and respiration, which take up or release 1 mole of DIC for every mole of organic carbon (CH_2_O) produced or decomposed with little influence on seawater TA. In contrast, the inorganic carbon cycle (NCC) is dominated by CaCO_3_ precipitation and dissolution, which alter TA and DIC in a ratio of 2:1 for every mole of CaCO_3_ precipitated or dissolved. Photo credit: Yuna Zayasu, OIST. (B) Depending on the relative contribution from different metabolic processes, the resulting change in TA and DIC influences seawater pH differently (colored contours). Photosynthesis and CaCO_3_ dissolution increase seawater pH while respiration and CaCO_3_ precipitation decrease pH. If NCP and NCC are closely balanced (i.e., TA-DIC slope ~1), there is little change in seawater pH owing to net reef metabolism. This is because the slope of pH isolines within the normal oceanic concentration of seawater TA and DIC are close to 1. Therefore, when the slope of the TA-DIC vector is different from 1 the pH isolines are crossed and seawater pH can be altered considerably. The calculations for pH at each TA and DIC value assume constant temperature (25°C) and salinity (35). (C) Conceptual schematic of the biogeochemical and metabolic function of coral reefs. Net CaCO_3_ precipitation (+NCC, green area) vs. net dissolution (-NCC, pink/red area); net autotrophy (+NCP) vs. net heterotrophy (-NCP), and different TA-DIC slopes, as well as the resulting changes in reef seawater pH (pH_r_) relative to the open ocean (pH_o_) under constant salinity and temperature conditions.

A coral reef’s metabolic influence on seawater chemistry is reflected as the rhythmic, diel and seasonal cycles of seawater TA and DIC (i.e., the ‘metabolic pulse’). Because changes in TA and DIC largely reflect coral reef metabolism (Fig 1), they can provide useful metrics that indicate perturbations to coral reefs on organismal, community, and ecosystem scales. One such metric is the TA anomaly between reef and source seawater, which we term the net calcification potential. Comparing reef TA concentrations to surrounding open ocean or source seawater (ΔTA) provides a direct indication of whether a reef is net calcifying (TA depletion) or dissolving (TA repletion) [12, 29–31], although to determine the exact rate of CaCO_3_ precipitation/dissolution also requires estimates of the seawater residence time and volume of water above the reef. However, changes in ΔTA monitored over time at individual reefs could indicate shifts in NCC assuming there are no major concurrent changes to reef hydrodynamics and seawater residence time. Therefore, monitoring the net calcification potential of coral reefs (i.e., ΔTA) through time could be critical in guiding conservation and management efforts by contributing to a better understanding of a reef’s ability to deposit CaCO_3_.

Another potential metric of reef biogeochemistry derived from TA and DIC measurements is the slope of TA-DIC regressions, which reflects the balance of NCP and NCC, such that in a system dominated by NCP the slope approaches 0 while in a system dominated by NCC the slope approaches 2. Because of these properties, graphical vector analysis of seawater TA and DIC (e.g., the TA-DIC slope or ΔTA:ΔDIC) quantifies the relative balance of CaCO_3_ to organic carbon fluxes [29, 32, 33], which can directly relate to properties such as community composition (e.g., calcifying vs. non-calcifying organisms) and net reef metabolic status (Fig 1) [34, 35]. The balance of NCP and NCC also reflects how a reef modifies seawater pH, and thus, whether net reef metabolism elevates or suppresses reef pH relative to the open ocean. Graphical vector analysis of TA and DIC [33] has been successfully used to characterize reefs as sources or sinks of CO_2_ to the atmosphere [32], to characterize the dominant processes responsible for the modification of seawater chemistry across different reef habitats [9, 23], and to demonstrate how coral bleaching and outbreaks of predatory starfish resulted in a reef-wide decrease in net community calcification [29]. Consequently, the TA-DIC vector approach is a powerful tool that can be used to compare the balance of NCP and NCC on different coral reefs, and potentially, a simple and effective monitoring tool of changes to coral reef community metabolism arising from ocean warming, ocean acidification, and other perturbations over time.

Herein, we gathered seawater TA and DIC data from 27 temporal or spatial sampling expeditions covering 23 global coral reef locations in all major ocean basins (S1 Fig). The studies ranged in scope from one day at a single reef location to multiple years covering extensive spatial scales (>10 km^2^). Based on these studies we evaluate the current net calcification potential and the relative balance of NCP and NCC across a global selection of coral reefs; we qualitatively assess whether TA-DIC slopes can be a useful metric for monitoring changes in reef biogeochemistry due to anthropogenic perturbations; we detail how seawater carbonate chemistry measurements can better inform our understanding of coral reef susceptibility to future ocean acidification; and, we propose that future studies need to take an interdisciplinary approach combining ecological, biogeochemical, and physical measurements to develop a mechanistic understanding of coral reef ecosystems.

## Materials and methods

Surface seawater TA, DIC, temperature, and salinity data were gathered from 23 coral reef locations from previously published and unpublished sources (S1 Fig, S1 Table) [14, 20, 29, 36–51]. Total alkalinity and DIC were directly measured in most studies. However, there were three studies that relied on TA and pH measurements to calculate DIC concentrations [37, 38, 45], which adds some additional uncertainty to estimates of DIC (<1%) [52, 53]. In most studies, seawater sampling, preservation, and carbonate system analyses were performed according to the Guide to Best Practices [53]. For exact details on sample collection, preservation, and analysis at each site the reader is referred to the citations in S1 Table. Although these studies represent a broad range of reef environments with different biological (e.g., biomass and community composition), physical (e.g., depth and residence time), and geochemical properties, as well as observations made on different temporal and spatial scales, the net calcification potential and the balance of NCP and NCC offer direct assessments of reef biogeochemical function within the scope of each study. Thus, it is beneficial to maximize the range of studies to assess the diversity in reef biogeochemical function, although at present it may not be possible to fully assign exact attribution to any observed differences. However, such differences may serve as important guidelines for future studies.

The TA anomaly (ΔTA) was calculated as the difference between reef and offshore TA values (i.e., reef TA—offshore TA), with negative values representing TA depletion (+NCC) and positive values representing TA repletion (-NCC). When offshore TA concentrations could not be obtained from the individual studies, they were estimated from GLODAP gridded data [54]. For TA-DIC regression analysis, the TA and DIC data from each study were grouped together regardless of sampling time and season. Type II linear regression analysis was performed on TA and DIC data in Matlab using the lsqfitma.m MODEL-2 least squares fit code (http://www.mbari.org/staff/etp3/regress.htm). The only exception was for data from Eilat Reef in the Red Sea which were grouped according to the year it was collected due to large changes in the offshore TA concentrations between different years [48]. The relative % influence of NCP on changes in DIC was calculated according to the following equation;

$$\%\;\text{NCP}=\left(1-\frac{{\text{m}}_{\text{TA}-\text{DIC}}}{2}\right)\times 100$$ (1)

where m_TA-DIC_ is the slope of the TA-DIC vector.

**Fig 2:**
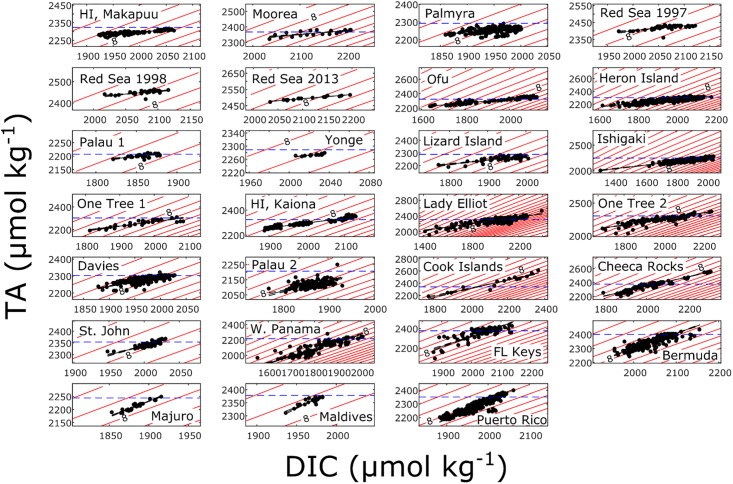
Seawater TA and DIC data from 23 coral reef locations around the world in increasing order of TA-DIC slope. The black line is the Type II linear regression of the data. Red lines are pH isolines at 0.1 units increments with the 8.0 pH isoline indicated for reference. pH contours were calculated using the average seawater temperature and salinity of each dataset. The blue dashed line in each panel is the approximate TA of the offshore seawater determined as described in the text.

For all calculations, TA and DIC data were not normalized to salinity because of a wide range of values between locations, which could potentially introduce artifacts into the data. Regardless, normalizing the data had a minimal impact on both the average ΔTA values and TA-DIC slopes (S2 and S3 Figs). To generate the pH contour lines in Fig 2, pH was calculated on the total H^+^ scale using the Matlab code equic.m [55] at the average seawater temperature and salinity of each site. Each dataset was determined to be either; (1) temporally based (temporal) if sampling was conducted over a small spatial scale (<10km^2^) and changes in TA and DIC were predominantly measured over time, or (2) spatially based (spatial) if the sampling protocol involved sampling across a large spatial scale (>10km^2^).

## Results and discussion

### The current state of net ecosystem calcification

**Fig 3:**
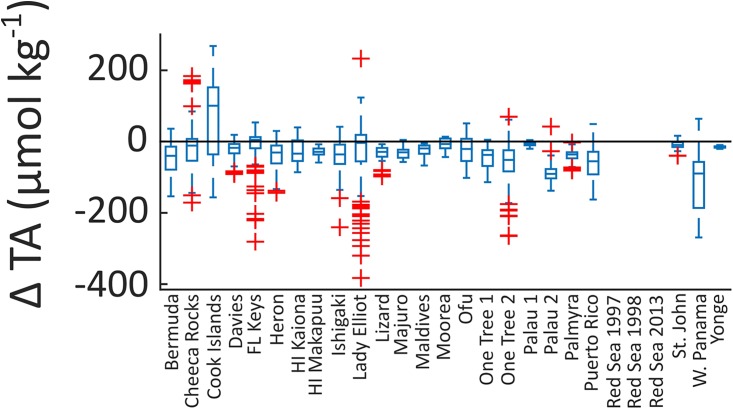
Net calcification potential measured as anomalies between open ocean and coral reef TA concentrations (ΔTA) at each location. Negative values are lower TA concentrations within the reef and represent net CaCO_3_ precipitation (+NCC), while positive values are higher TA concentrations within the reef and represent net CaCO_3_ dissolution (-NCC). Edges of the box are the 25^th^ and 75^th^ percentiles, the line within each box is the median, the whiskers represent the most extreme data points that are not outliers, and the red + symbols are outliers.

Most of the coral reefs in this study showed extensive depletion of TA relative to adjacent open ocean values (negative ΔTA) for the majority of observations (Figs 2 and 3). The average ΔTA within individual reef systems ranged from -114 to +68 μmol kg^-1^ (S1 Table). The only reef that had a positive average ΔTA was Muri Lagoon in the Cook Islands, which was influenced by the input of high TA groundwater [37]. The lowest average ΔTA (-114 μmol kg^-1^) was observed in Western Panama (Pacific), which could be due to high rates of NCC and/or long residence times assuming that no other processes such as upwelling or freshwater input from rain or runoff affected seawater TA [44]. On average, the observed ΔTA relative to offshore was -36 ± 26 μmol kg^-1^ for all locations, with the Cook Island dataset excluded because of substantial groundwater input [37]. This is direct evidence that the reefs in this study, on average, are currently maintaining net calcification. However, most reefs also appeared to undergo net CaCO_3_ dissolution at times (mainly at night) as indicated by positive ΔTA values. Therefore, when evaluating ΔTA it is critical to devise a sampling strategy that adequately assesses times when both net CaCO_3_ production and dissolution are more likely to affect water column TA measurements (e.g., midday, early morning, or night).

The proportion of positive ΔTA observations at each reef site (indicative of times of net dissolution or negative NCC) ranged from 0 to 67% (S1 Table). Between oceanic regions, the average percent occurrences of net dissolution ranged from 25 ± 22% for reefs in the Atlantic Ocean, 11 ± 16% within the Great Barrier Reef (GBR), 19 ± 22% throughout the rest of the Indo-Pacific, and 6% in Western Panama (we were unable to calculate ΔTA for the Red Sea dataset as the TA of the source water was highly variable) (Figs 2 and 3). With the exception of Lady Elliot Island, reefs in the GBR had relatively low occurrences of net dissolution (≤11%). Importantly, reefs in the GBR were all sampled over complete diel cycles and had no temporal sampling bias, which would not be the case for reefs sampled only during the daytime. Given the large variability within each region, it can be concluded that incidents of net dissolution are mostly related to local reef conditions rather than regional differences. Net dissolution mainly occurs at night coincident with net respiration, high CO_2_ concentrations, and low rates of gross calcification [12, 14, 36, 45]. Net dissolution can also occur on a seasonal basis and was observed during winter months in both Bermuda [31, 56] and Florida [46]. In fact, 34% and 58% percent of the observations from Cheeca Rocks and the Florida Keys, respectively, indicated net dissolution, which was hypothesized to be caused by seasonally driven net respiration and low rates of calcification during winter [46].

Based on results from this and previous studies, monitoring changes in ΔTA over time serves as a relatively easy, highly sensitive, and instantaneous indicator of coral reef net calcification under both natural and anthropogenic environmental change. However, it is important to recognize that ΔTA doesn’t provide a quantitative measurement of NCC without knowing seawater residence times and volume. A number of approaches have been used to estimate seawater residence time for different reefs (e.g., using salinity anomalies [48, 57, 58], radioactive tracers such as ^7^Be [46, 57], and direct measurements of water movement and currents [59, 60]) but currently these estimates remain limited to a few locations and are generally associated with large uncertainty [57]. However, recent advances in analytical capabilities to measure various isotopes and/or tracers in seawater combined with access to hydrodynamic modeling tools may assist in characterizing the residence time more precisely and at a larger number of reefs in future studies.

### The relative balance of NCP and NCC

**Fig 4:**
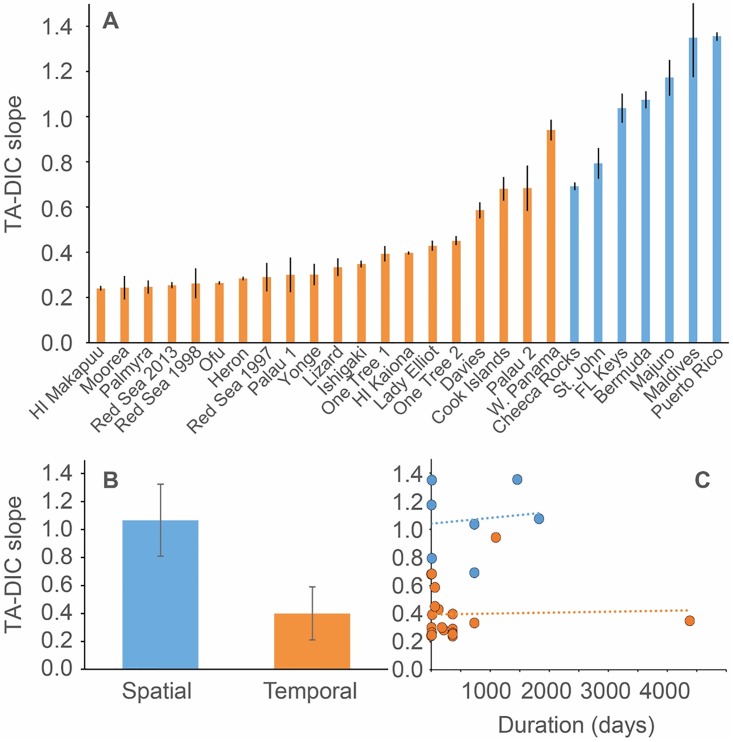
The influence of spatial and temporal sampling scales on TA-DIC slopes. (A) The TA-DIC slopes from the different coral reef locations calculated using a Type II linear regression (*see* also S1 Table). Error bars are ±1 SD of the slope. The colors indicate whether the sampling protocol had a significant spatial component (>10 km^2^; blue) or was predominantly sampled over time at one location (orange). (B) Comparison of the average TA-DIC slope (± standard deviation) of all temporally and spatially sampled reefs. (C) TA-DIC slopes as a function of the duration of each study in days grouped by spatial (blue) and temporal (organge) sampling protocols.

The relative influence of NCP on changes in DIC concentrations at the different reef sites ranged from 32% to 88% as indicated by TA-DIC slopes ranging from 0.24 to 1.36 (Figs 2 and 4A, S1 Table). Overall, reefs in the Atlantic Ocean had relatively high TA-DIC slopes compared to most other reefs, ranging from 0.69 (Cheeca Rocks) to 1.36 (Puerto Rico), while Indo-Pacific reefs (including the GBR) had a larger range from 0.24 (Hawaii) to 1.35 (Maldives). Importantly, the relatively higher TA-DIC slopes in Atlantic compared to Indo-Pacific reefs was strongly influenced by the spatial scale of the studies. In general, studies covering a larger spatial scale (>10km^2^) had significantly higher TA-DIC slopes compared to studies characterizing seawater carbonate chemistry at only one site (p<0.001; Wilcoxon test; Fig 4B). At larger spatial scales, carbonate chemistry measurements represent an integrated signal of multiple reef habitats and communities rather than the signal of one localized community. At these scales, the trophic status of coral reefs is most likely closely balanced (i.e., NCP = 0) [61, 62], and thus, TA-DIC slopes are more strongly influenced by NCC compared to localized studies that are more strongly influenced by high frequency variations in NCP. Consequently, when comparing the relative balance of NCP and NCC between different reefs it is important to only compare studies of similar spatial coverage. In contrast, the duration of the studies appeared to have no influence on TA-DIC slopes (Fig 4C). Based on these considerations, most of the temporally based studies were dominated by NCP with the percent influence of NCP on DIC changes ranging from 53% to 88% (S1 Table). Of the temporally based studies, sites in the Red Sea were most strongly dominated by NCP (87% ± 0.9%; average ± SD), while sites in the GBR (80% ± 5%) and the rest of the Indo-Pacific (81% ± 9%) had a relatively lower and similar average percent influence of NCP comprising a larger range. Within the spatially based studies, Cheeca Rocks (65%) and St. John (60%) were most strongly influenced by NCP while the Maldives (32%) and Puerto Rico (32%) were least influenced. Unfortunately, all of the Atlantic based studies were classified as spatial, while the majority of the Pacific based studies were classified as temporal, making any comparisons between the ocean basins tenuous.

It might be hypothesized that reefs with higher TA-DIC slopes (i.e., higher %NCC) would also have a greater depletion of TA due to high rates of NCC. Notably, there was no apparent correlation between average ΔTA or percent occurrences of dissolution and TA-DIC slopes within our dataset (S4 Fig). This qualitatively indicates that the relative balance of NCP and NCC (i.e., organic vs. inorganic metabolism) is not directly related to the production of CaCO_3_, recognizing the caveat that ΔTA is only a quasi-quantitative estimate of calcification (i.e., even reefs with a relatively small depletion of TA may produce large amounts of CaCO_3_ if the residence time is short). Most importantly, the results show that even reefs and/or reef habitats dominated by NCP still undergo substantial net calcification. This is supported by visual and quantitative observations of reefs with low TA-DIC slopes (e.g., Heron and One Tree Islands) that have extensive and healthy coral populations and high rates of NCC [14, 45, 63]. Differences in TA-DIC slopes, and subsequently NCP:NCC, could be related to differences in benthic community composition, including the percent cover of calcifying vs. non-calcifying organisms [9, 32, 43]. Regrettably, the current TA-DIC dataset was not associated with detailed benthic community composition characterizations or estimates of chemical footprint dimensions for all study sites. Consequently, it was not possible to evaluate how the observed balance of NCP and NCC and variation in carbonate chemistry were related to *in situ* benthic communities. However, a recent short-term (24 h) mesocosm experiment with diverse benthic communities from Hawaii illustrated that the TA-DIC slope, TA anomaly, and seawater pH varied predictably according to community composition and relative rates of NCP and NCC [34]. These results demonstrate that different calcifying communities can have similar TA-DIC slopes (e.g., corals, calcifying algae, and sand), but radically different TA anomalies related to differences in the absolute rates of NCC. This adds insight into the lack of correlation between average ΔTA and TA-DIC slopes from the *in situ* data (S2 Fig), and indicates that reef sites with similar TA-DIC slopes may have different benthic community composition with different calcification capacities. Alternatively, recent work from Mo’orea has also shown TA-DIC slopes to be taxa-specific, suggesting that a community’s TA-DIC slope may not only be dependent on the overall percent cover of calcifiers, but also the relative abundance and diversity of these calcifiers [35]. On an ecosystem scale, TA-DIC slopes reveal the relative balance between NCP and NCC, and observations over time at a single reef location will be useful for detecting local changes in the percent cover of calcifiers and shifts in biogeochemistry due to environmental perturbations. Changes in the TA-DIC slope over time will also be affected by other processes besides coral calcification such as changes in CaCO_3_ sediment dissolution [11, 64] and chemical bioerosion [65, 66], making it a true indicator of ecosystem level response to climate change.

### A coral reef’s influence on seawater pH

The local modification of TA and DIC determines how seawater pH changes within that reef. Reef sites with the lowest TA-DIC slopes and the largest absolute change in DIC experienced the greatest variability in seawater pH (Figs 1 and 2). In contrast, reefs with balanced NCP and NCC ratios experienced less variability in pH. The reason for this is that reefs with a TA-DIC slope close to 1 change seawater chemistry along isolines of constant pH while reefs with a slope significantly different from 1 cross isolines of constant pH (Figs 1 and 2) [9]. This is because the slope of pH isolines within the normal oceanic concentration of seawater TA and DIC are close to 1. Consequently, if the net metabolism of coral reefs remains unchanged under future ocean acidification, reefs with a TA-DIC slope substantially different from 1 could exacerbate or counteract the effects of ocean acidification depending on the reef’s overall trophic status (i.e., net autotrophic or heterotrophic) (Fig 1C). The modification of pH within individual coral reefs may relate to different susceptibilities and resistance, as variability in seawater pH has been shown to modulate the response of some corals to ocean acidification [67]. Recent studies have shown that reductions in coral calcification due to decreasing pH may be tempered under oscillating compared to constant carbonate chemistry conditions [27, 28], while other studies have showed no enhanced tolerance to ocean acidification by corals in low compared to highly variable pH conditions [26, 68]. Furthermore, while the response of coral calcification to a reduction in average pH has been shown to be robust [69], there are examples of corals thriving in low pH environments [70, 71], including deep water corals thriving in seawater with a Ω_ar_ well below 1 [72]. The complex and nuanced responses of corals to seawater pH make it critical to evaluate how seawater pH changes currently, and will change into the future across a diverse array of coral reef systems. It is important to highlight that seawater pH variability is localized within coral reef ecosystems and dependent on a number of factors such as water column depth, residence time, community composition, etc. For example, the deeper areas of a reef (e.g., reef slopes where a large portion of calcification occurs) will most likely experience conditions similar to the open ocean rather than experiencing localized variability in carbonate chemistry which is most pronounced in shallow areas such as on rim reefs and reef flats. This work offers an initial, baseline examination of local carbonate chemistry in a global sample of coral reefs, knowledge of which is critical to predicting future changes due to ocean acidification within individual reefs.

### Concluding remarks

The results of this study clearly demonstrate that there is a large amount of variability in the carbonate chemistry overlying coral reef communities and ecosystems throughout the world, which could affect how individual reefs respond to global change. While some reefs experienced instances of net CaCO_3_ dissolution, all reefs in this study were net calcifying for the majority of observations. This indicates that, from a chemical perspective, the reefs in this study are currently maintaining positive CaCO_3_ production critical for the maintenance of reef structure and function (Fig 3). However, these chemical measurements do not tell us if net calcification is able to offset any mechanical CaCO_3_ erosion and off-reef transport. It is important to emphasize that, despite the lack of reef scale estimates of absolute rates of net calcification, ΔTA serves as an important indicator of whether a reef is net calcifying or net dissolving, and can show how relative rates of NCC vary over time. When using ΔTA as an estimate of coral reef net calcification potential it is also important to consider other biogeochemical processes that could alter seawater TA concentrations such as submarine groundwater discharge [37]. The observed differences in the relative balance of NCP and NCC within these systems reflect inherent differences in net reef metabolism (i.e., net autotrophic, heterotrophic, calcifying, or dissolving) [12, 14, 49, 62, 73], the benthic community composition [22, 23], organic and inorganic carbon inputs from rivers, groundwater, and/or advection from offshore [74, 75], as well as the spatial scale of the study (Figs 2 and 4). Similar to ΔTA measurements, TA-DIC slopes and the relative balance of NCP and NCC can provide important metrics of reef metabolism and function over time [29, 32, 43], but it is critical that the spatial scale of the data is carefully considered.

Additional efforts integrating TA and DIC measurements with traditional surveying techniques and/or remote sensing at different scales will be important to fully link the chemical seawater signal with coral reef benthic community structure, function, and health. Currently, coral reef community composition and health are mainly monitored visually by SCUBA divers using transects and, more recently, photographic methods which are typically labor intensive, expensive, and partly reliant on subjective interpretation. Recent advances in remote sensing, imaging technologies, and machine learning will greatly improve these visual observations [76, 77]. However, these approaches do not provide direct information about the biogeochemical processes and elemental cycling of coral reefs, which are becoming increasingly important to monitor. Ideally, coral reef monitoring programs should include information on both community composition and quantitative biogeochemical data assessing the organic and inorganic carbon cycles. Carbonate chemistry measurements are becoming increasingly automated [78, 79], which will improve the feasibility and practicality of monitoring the ‘metabolic pulse’ of coral reefs and other marine ecosystems. Furthermore, the use of autonomous vehicles, satellite products, and perhaps even citizen scientists [80] will facilitate data collection over larger spatial and longer temporal scales.

In conclusion, this study offers an overview of the metabolic function for a range of coral reefs at different scales that warrants a more sophisticated and interdisciplinary approach in future studies. This synthesis of carbonate chemistry within a selection of the world’s coral reefs provides a framework for developing hypotheses aimed at addressing the mechanistic attribution to observed differences in the ‘metabolic pulse’ and serves as a critical baseline for building an understanding of how ocean acidification and other perturbations will impact individual coral reef ecosystems. Results herein confirm, based on global observations, that seawater pH within reef systems will not only be determined by how open ocean CO_2_ chemistry is changing, but by complex interactions between benthic metabolism, local hydrodynamics, terrestrial inputs, and any changes in community composition [20, 21, 31]. It is also apparent that the carbonate chemistry within individual reef systems varies greatly across the globe, and even spatiotemporally within individual reefs and habitats. Importantly, knowing the current balance of NCP and NCC within a coral reef already gives us an idea of which reefs experience greater variability and extremes in pH, and how the chemistry within each reef will change as a result of changing community structure, net reef metabolism, and ongoing ocean acidification (e.g., [20]). This knowledge is critical to accurately determine the biological and geochemical effects of ocean acidification on reef systems. Observed differences in the TA-DIC slope between different reefs, and thus, their ability to exacerbate or alleviate ocean acidification could translate into different susceptibilities and resistance to environmental perturbations. If so, monitoring changes in ΔTA and the TA-DIC slope through time will provide valuable insights into the function and health of coral reefs that may be critical in guiding conservation efforts aimed at maximizing the success of these ecosystems.

## Supporting information

S1 Table Data from the global TA-DIC coral reef analysis. Columns are (1) location of each study; (2) ocean basin the study was conducted in; (3) slope of the TA-DIC vector; (4) percent influence of NCP on changes in DIC; (5) R^2^ of the TA-DIC slope; (6) slopes of TA-DIC vectors normalized to the average salinity at each site (nTA-nDIC); (7) mean TA anomaly (ΔTA; μmol kg^-1^) at each site; (8) percent occurrences of dissolution (%D) at each site; (9) TA of the open ocean endmember used to calculate ΔTA and %D in μmol kg^-1^; (10) study type, classified as either temporal (T) if the study was conducted mainly at one spot over time or spatial (S) if greater than a 10 km^2^ area was sampled; (11) total number of samples in each study; (12) length of each study; and (13) citation where the data was taken from.
(XLSX)

S1 Fig Map of the coral reef sites used in this study. Some locations were combined because there was not enough spatial resolution to show as two distinct points. The colors and symbols indicate whether the reefs are in the Atlantic (green circles), Great Barrier Reef (red squares), Indo-Pacific (blue triangles), and other (grey diamonds) regions.
(TIF)

S2 Fig Regression of non-normalized and salinity normalized TA-DIC slopes. To calculate the salinity normalized slope (nTA-nDIC), TA and DIC data were normalized to the average salinity of each site.
(TIF)

S3 Fig Regression of average ΔTA using non-normalized and salinity normalized TA data. For ΔnTA data were normalized to the average salinity of each reef site.
(TIF)

S4 Fig The percent occurrence of net dissolution (red squares) and the average depletion of TA relative to offshore (ΔTA; blue circles) versus the TA-DIC slope from each dataset. (TIF)
