# Supplementary figures and images for: Taking the metabolic pulse of the world’s coral reefs
Source: PLoS One. 2018 Jan 9;13(1):e0190872. doi: 10.1371/journal.pone.0190872 (PMC5760028; doi:10.1371/journal.pone.0190872)

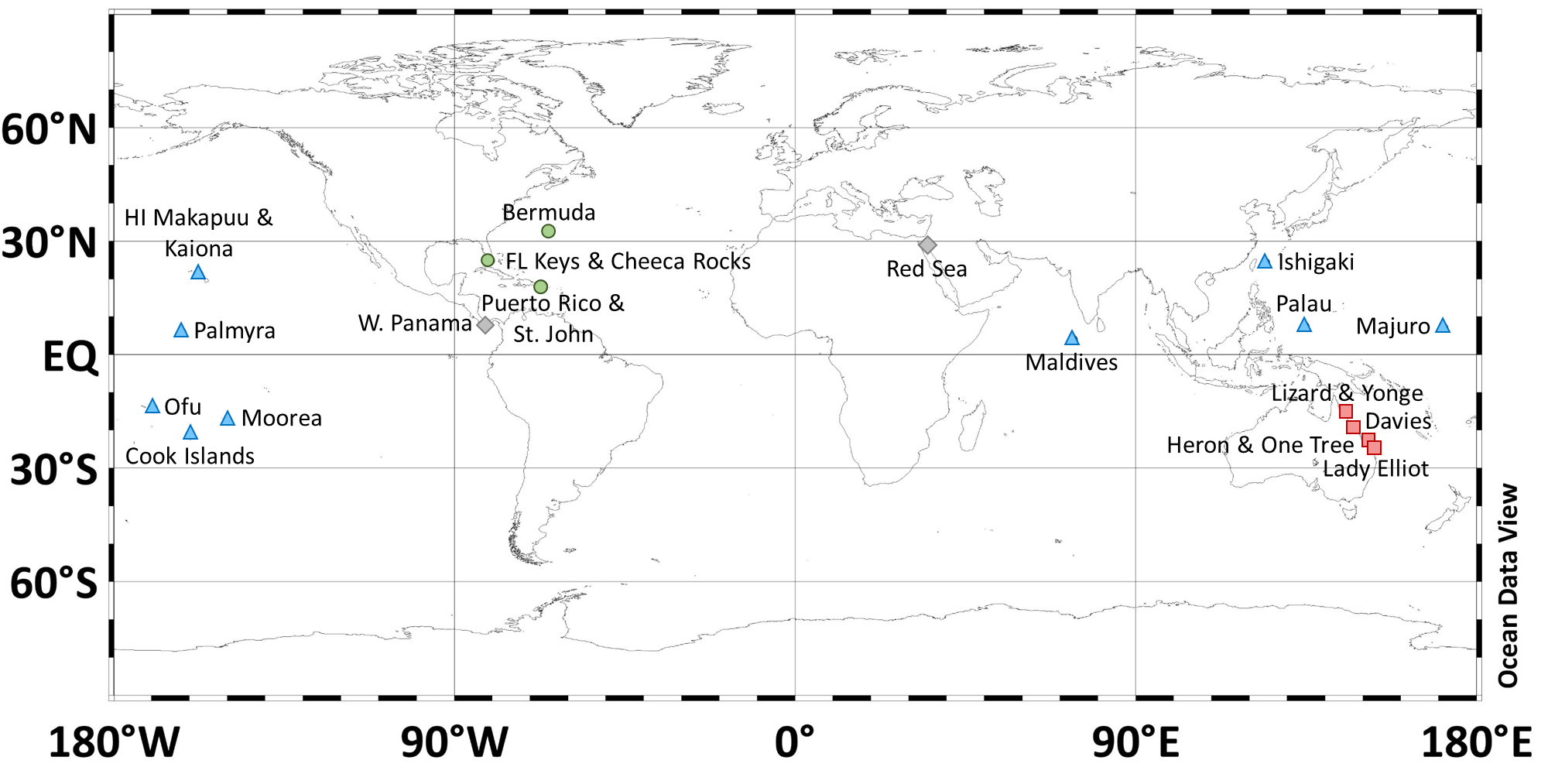

Supplement: S1 Fig — Some locations were combined because there was not enough spatial resolution to show as two distinct points. The colors and symbols indicate whether the reefs are in the Atlantic (green circles), Great Barrier Reef (red squares), Indo-Pacific (blue triangles), and other (grey diamonds) regions. (TIF) [file pone.0190872.s002.tif]

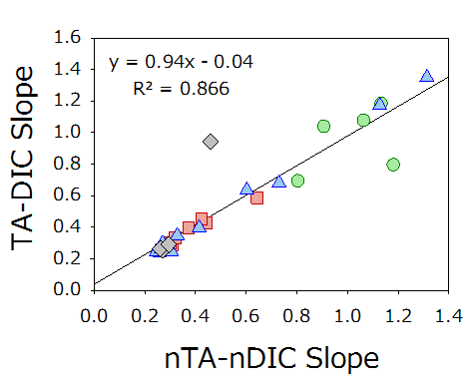

Supplement: S2 Fig — To calculate the salinity normalized slope (nTA-nDIC), TA and DIC data were normalized to the average salinity of each site. (TIF) [file pone.0190872.s003.tif]

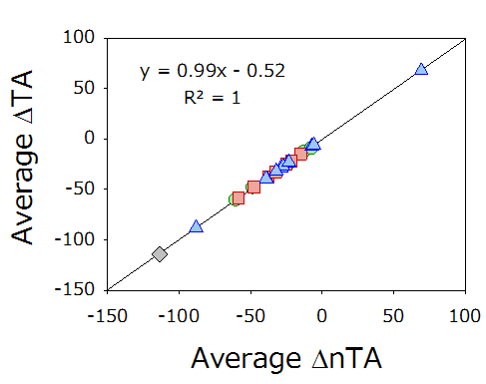

Supplement: S3 Fig — For ΔnTA data were normalized to the average salinity of each reef site. (TIF) [file pone.0190872.s004.tif]
